# Poster Session I - A152 IMPROVING ACCESS TO OUTPATIENT THERAPEUTIC PARACENTESIS: A QUALITY IMPROVEMENT APPROACH TO MINIMIZE MISSED SLOTS AND EMERGENCY DEPARTMENT RELIANCE.

**DOI:** 10.1093/jcag/gwaf042.152

**Published:** 2026-02-13

**Authors:** K Alseiari, M Alkhalifa, C Vezina, C Norris, J Masaoy, F Fadel, T Afzaal, M Q Khan, A Teriaky, K Qumosani, D Hudson

**Affiliations:** Western University Schulich School of Medicine & Dentistry, London, ON, Canada; Western University Schulich School of Medicine & Dentistry, London, ON, Canada; Western University Schulich School of Medicine & Dentistry, London, ON, Canada; Western University Schulich School of Medicine & Dentistry, London, ON, Canada; Western University Schulich School of Medicine & Dentistry, London, ON, Canada; Western University Schulich School of Medicine & Dentistry, London, ON, Canada; Western University Schulich School of Medicine & Dentistry, London, ON, Canada; Western University Schulich School of Medicine & Dentistry, London, ON, Canada; Western University Schulich School of Medicine & Dentistry, London, ON, Canada; Western University Schulich School of Medicine & Dentistry, London, ON, Canada; Western University Schulich School of Medicine & Dentistry, London, ON, Canada

## Abstract

**Background:**

Patients with cirrhosis and refractory ascites often require frequent large-volume paracentesis (LVP) to prevent symptomatic fluid accumulation and avoid emergency department (ED) visits or hospitalizations. At our tertiary care center, the outpatient paracentesis clinic offers six procedure slots weekly; however, baseline data showed underutilization, with 20–30% of slots remaining unfilled.

**Aims:**

To optimize outpatient paracentesis clinic utilization through targeted workflow interventions and to assess whether improved access correlated with reduced ED visits for paracentesis.

**Methods:**

This single-center pre-and post-intervention quality improvement (QI) at a tertiary-care liver program with a weekly outpatient paracentesis clinic (six slots per week). The baseline period (January–May 2024) established utilization patterns, followed by an intervention period (January–September 2025)

Change Idea #1: Tracking and development of individualized “dry weights” and standardized patient education to identify when paracentesis was not required. Nursing staff performed pre-appointment assessments 48–72 hours prior to scheduled procedures to confirm.

Change Idea #2: Implementation of an urgent-waitlist system to fill same-day cancellations resulting from patients at dry weight or without a safe paracentesis pocket.

Monthly clinic utilization and ED visit data were analyzed descriptively to assess trends before and after intervention.

**Results:**

During the baseline period (January–May 2024), 212 outpatient paracenteses were performed (mean 17.7 per month), with 20–30% of slots unfilled. Following the January 2025 intervention, throughput improved progressively. Between January and April 2025, 67 procedures were completed (mean 8.4 per month), and from May to September 2025, 109 were performed (mean 21.8 per month), a 23% increase from baseline. In the latest three months (July–September 2025), 69 procedures were completed (mean 23.0 per month), and unfilled slots declined to 11.9%, indicating sustained improvement and consistent scheduling. ED visit review (January–September 2025) showed higher use during periods of lower clinic throughput and fewer visits during months of optimal performance, demonstrating an inverse relationship between outpatient capacity and acute care demand.

**Conclusions:**

Implementation of structured QI measures—pre-confirmation, rapid slot substitution, and individualized dry-weight protocols—significantly improved outpatient paracentesis utilization and reduced unfilled capacity. Enhanced clinic efficiency correlated with a reduction in ED visits, supporting the role of proactive outpatient optimization in improving access, resource use, and patient outcomes in advanced liver disease.

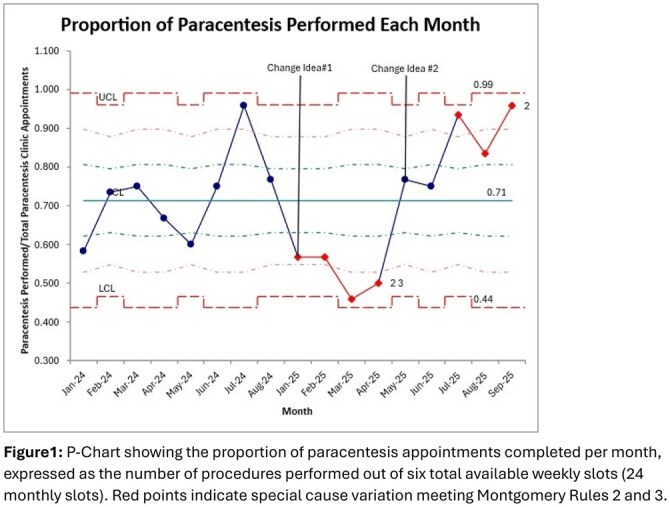

**Funding Agencies:**

None

